# Supplementary material for: Bounded contribution of human early visual cortex to the topographic anisotropy in spatial extent perception
Source: Commun Biol. 2024 Feb 13;7:178. doi: 10.1038/s42003-024-05846-x (PMC10864322; doi:10.1038/s42003-024-05846-x)
Supplement: Supplementary file 2 — Supplementary Information [file 42003_2024_5846_MOESM2_ESM.pdf]

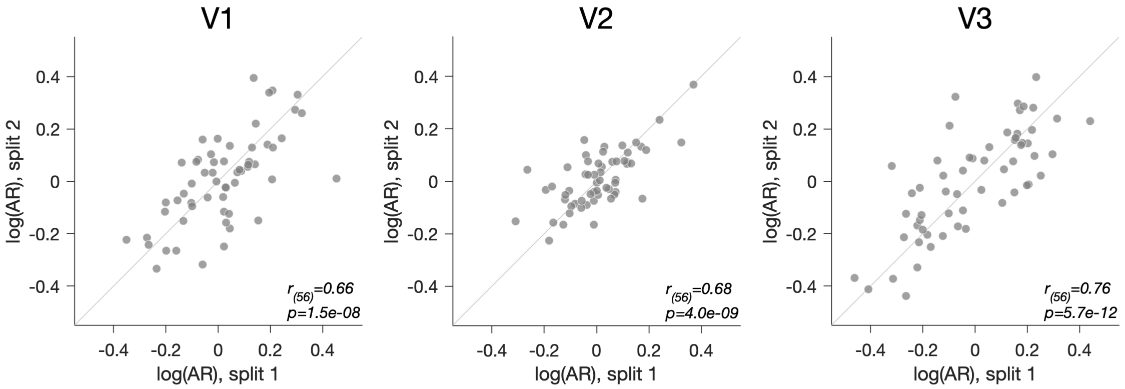

Supplementary Figure 1. Split-half reliability of the pRF anisotropy estimates for V1, V2, and V3 voxels. To measure the internal reliability of the aspect ratio (AR) estimates, we conducted a split-half reliability test. We randomly sampled half (4 cycles) of the total (8 cycles) BOLD time series data for each of the radial and tangential orientation conditions. Then, we estimated the pRFs and obtained the across-voxel averages of the log values AR for each of these two split halves. The values obtained from one half are plotted against the corresponding values obtained from the other half. The correlation coefficients were substantially high ($r_{(56)}$=0.66 for V1, 0.68 for V2 and 0.76 for V3; voxel selection criterion $r > 0.25$ was applied). We opted not to split the entire time series into the early and late halves because large-scale background co-fluctuations are shared by an entire population of neurons regardless of whether individual neurons' stimulus preferences match incoming visual input or not (Jack et al., 2006; Donner et al., 2008; Sirotin and Das, 2009; Choe et al., 2014; Ryu and Lee, 2018). Pearson correlation *r* and two-tailed *p*-values are shown in the lower right corner. Dots, 29 observers X 2 orientation conditions.

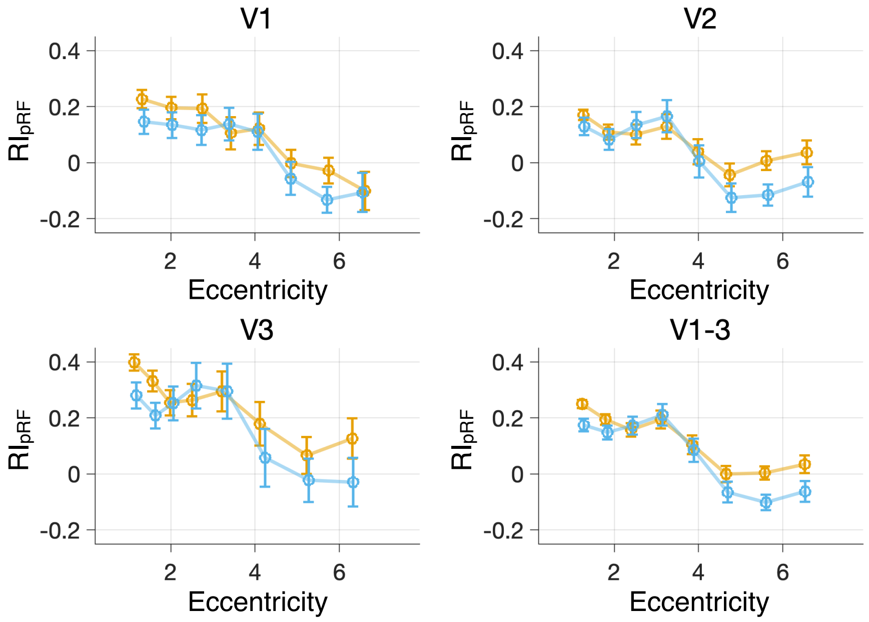

Supplementary figure 2. The radial bias indices of the pRF anisotropy (RI_{pRF}) plotted against their eccentricity positions for the data from the individual visual areas V1, V2, V3 and the data averaged across the areas. We grouped voxels into eight eccentricity bins of equal sizes based on their pRF eccentricity. At each eccentricity bin, we computed the across-voxel averages of the pRF eccentricity and of RI_{pRF} values. The pRF data were aggregated from 29 observers. Dot and error bar, across-voxel means and 95% CI. Orange and blue colors, the radial and tangential orientation conditions.

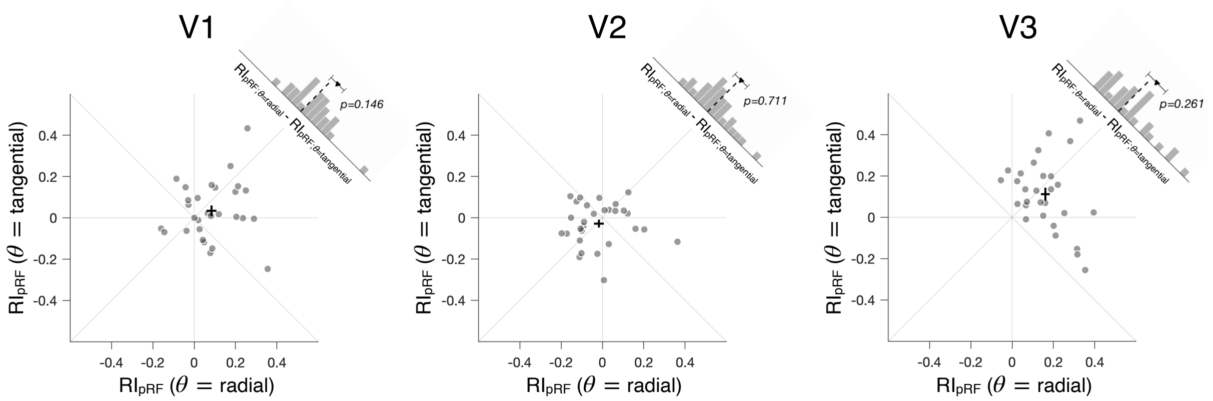

Supplementary figure 3. Comparison of the radial bias indices of the pRF anisotropy between the radial, $RI_{\mathrm{pRF}}(\theta = \mathrm{radial})$, and tangential, $RI_{\mathrm{pR}F}(\theta = \mathrm{tangential})$, orientation conditions for V1, V2, and V3. Here, RI_{pRF} is quantified as the logarithm of the radial-to-tangential aspect ratio of pRF spatial extent: $RI_{\mathrm{pRF}} = \log\,(\sigma_{\mathrm{radial-axis}}/\,\sigma_{\mathrm{tangential-axis}})$. The histogram in the upper-right corner of each panel shows the across-correlation distribution of differences in RI_{pRF} values between the two orientation conditions (2-tailed paired t-test; t_{28}=1.50, p=0.146, 95% CI=[-0.018°, 0.118°] for V1; t_{28}=0.37, p=0.711, 95% CI=[-0.051°, 0. 074°] for V2; t_{28}=1.14, p=0.261, 95% CI=[-0.038°, 0.136°] for V3). Dots, 29 individuals; Black crosshairs, across-individual means and their standard errors.

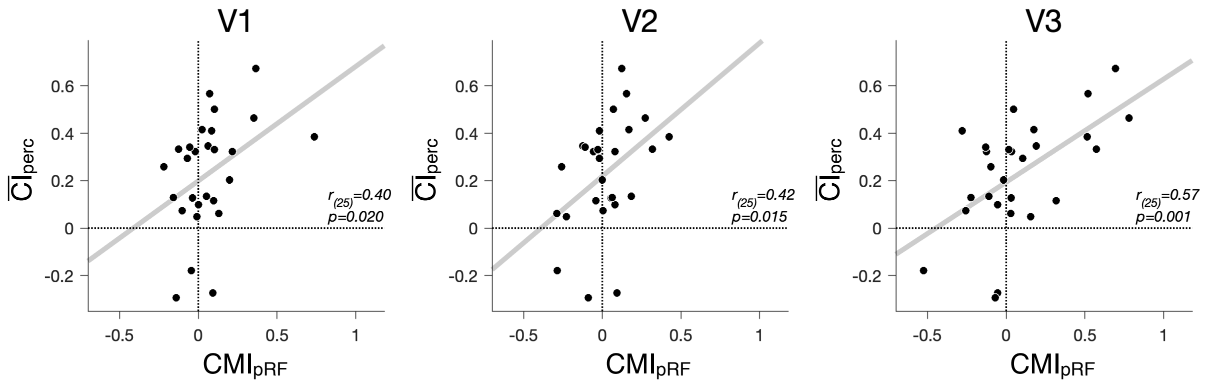

Supplementary figure 4. Across-individual co-variations between the influences of co-axiality on the pRF and perceptual anisotropies. The mean of co-axial bias indices of the perceptual anisotropy ($\overline{CI}_{\mathrm{perc}}$) of 27 individual observers are plotted against their co-axial modulation indices of the pRF anisotropy (CMI_{pRF}). The $\overline{CI}_{\mathrm{perc}}$ values were significantly regressed onto the CMI_{pRF} values for V1, V2, and V3, as depicted by linear regression line and Pearson correlation $r_{(25)}$ with one-tailed p values. The same equations described in the top and bottom panels in Figure. 6c were applied to compute CMI_{pRF} and $\overline{CI}_{\mathrm{perc}}$. Dots, 27 individuals.

References

Choe KW, Blake R, Lee SH (2014) Dissociation between neural signatures of stimulus and choice in population activity of human V1 during perceptual decision-making. Journal of Neuroscience 34:2725–2743.

Donner TH, Sagi D, Bonneh YS, Heeger DJ (2008) Opposite Neural Signatures of Motion-Induced Blindness in Human Dorsal and Ventral Visual Cortex. The Journal of Neuroscience 28:10298–10310.

Jack AI, Shulman GL, Snyder AZ, McAvoy M, Corbetta M (2006) Separate Modulations of Human V1 Associated with Spatial Attention and Task Structure. Neuron 51:135–147.

Ryu J, Lee SH (2018) Stimulus-Tuned Structure of Correlated fMRI Activity in Human Visual Cortex. Cerebral Cortex 28:693–712.

Sirotin YB, Das A (2009) Anticipatory haemodynamic signals in sensory cortex not predicted by local neuronal activity. Nature 457:475–479.
