# Supplementary material for: Bounded contribution of human early visual cortex to the topographic anisotropy in spatial extent perception
Source: Commun Biol. 2024 Feb 13;7:178. doi: 10.1038/s42003-024-05846-x (PMC10864322; doi:10.1038/s42003-024-05846-x)
Supplement: Supplementary file 5 — Reporting Summary [file 42003_2024_5846_MOESM5_ESM.pdf]

nature portfolio

Corresponding author(s): Sang-Hun Lee

Last updated by author(s): Aug 9, 2023

Reporting Summary

Nature Portfolio wishes to improve the reproducibility of the work that we publish. This form provides structure for consistency and transparency in reporting. For further information on Nature Portfolio policies, see our Editorial Policies and the Editorial Policy Checklist.

Statistics

For all statistical analyses, confirm that the following items are present in the figure legend, table legend, main text, or Methods section.

n/a	Confirmed	
☐	☒	The exact sample size (*n*) for each experimental group/condition, given as a discrete number and unit of measurement
☐	☒	A statement on whether measurements were taken from distinct samples or whether the same sample was measured repeatedly
☐	☒	The statistical test(s) used AND whether they are one- or two-sided<br>*Only common tests should be described solely by name; describe more complex techniques in the Methods section.*
☒	☐	A description of all covariates tested
☒	☐	A description of any assumptions or corrections, such as tests of normality and adjustment for multiple comparisons
☐	☒	A full description of the statistical parameters including central tendency (e.g. means) or other basic estimates (e.g. regression coefficient) AND variation (e.g. standard deviation) or associated estimates of uncertainty (e.g. confidence intervals)
☐	☒	For null hypothesis testing, the test statistic (e.g. *F*, *t*, *r*) with confidence intervals, effect sizes, degrees of freedom and *P* value noted<br>*Give P values as exact values whenever suitable.*
☒	☐	For Bayesian analysis, information on the choice of priors and Markov chain Monte Carlo settings
☒	☐	For hierarchical and complex designs, identification of the appropriate level for tests and full reporting of outcomes
☐	☒	Estimates of effect sizes (e.g. Cohen's *d*, Pearson's *r*), indicating how they were calculated

Our web collection on statistics for biologists contains articles on many of the points above.

Software and code

Policy information about availability of computer code

Data collection	The data from human experiments was acquired using the codes written in MATLAB in conjunction with PsychToolbox (Brainard, 1997) on a Macintosh computer.
Data analysis	We used SPM8 (Friston et al., 1996; Jenkinson et al., 2002) to correct slice-timing and motion of all functional EPI images. Then, we aligned the functional data from all scans to the T1-w anatomical image using the mrTools analysis package (doi: 10.5281/zenodo.1299483). To create a flattened representation of the occipital lobe, we constructed the cortical surface using Freesurfer (Dale et al., 1999; Fischl et al., 1999). We segmented the inner and outer layers of the gray matter using Jonas Larsson's SurfRelax. Finally, we used custom Matlab codes in mrLoadRet to display a flattened representation of the occipital cortex.

For manuscripts utilizing custom algorithms or software that are central to the research but not yet described in published literature, software must be made available to editors and reviewers. We strongly encourage code deposition in a community repository (e.g. GitHub). See the Nature Portfolio guidelines for submitting code & software for further information.

Data

Policy information about availability of data

All manuscripts must include a data availability statement. This statement should provide the following information, where applicable:

- Accession codes, unique identifiers, or web links for publicly available datasets
- A description of any restrictions on data availability
- For clinical datasets or third party data, please ensure that the statement adheres to our policy

Our preprocessed retinotopy and psychophysical data are publicly available on the Open Science Framework (https://osf.io/wrdt9/).

Research involving human participants, their data, or biological material

Policy information about studies with human participants or human data. See also policy information about sex, gender (identity/presentation), and sexual orientation and race, ethnicity and racism.

Reporting on sex and gender	Gender information was collected through self-reports for the study. In the fMRI data analysis, 15 females and 14 males were included. Twenty-seven of them also participated in the psychophysical experiment. For the psychophysical data analysis, 14 females and 13 males were included. This study did not expect any gender differences and did not conduct any analyses based on gender.
Reporting on race, ethnicity, or other socially relevant groupings	No data on ethnicity, race, or other social groupings were collected.
Population characteristics	The participants' age range was 20 to 30 years old, and all of them had normal or corrected-to-normal vision.
Recruitment	Participants were recruited through the advertisements in Seoul National University.
Ethics oversight	The Institutional Review Board of Seoul National University approved the conduct of this study.

Note that full information on the approval of the study protocol must also be provided in the manuscript.

Field-specific reporting

Please select the one below that is the best fit for your research. If you are not sure, read the appropriate sections before making your selection.

☐ Life sciences ☒ Behavioural & social sciences ☐ Ecological, evolutionary & environmental sciences

For a reference copy of the document with all sections, see nature.com/documents/nr-reporting-summary-flat.pdf

Behavioural & social sciences study design

All studies must disclose on these points even when the disclosure is negative.

Study description	This study uses a within-participant design and gathers sufficient data from each participant. Its goals are to quantitatively measure psychophysical responses while performing a circularity discrimination task and analyze cortical responses during a retinotopy mapping scan.
Research sample	The research sample primarily consists of graduate and undergraduate students from Seoul National University. Additional demographic information is provided above.
Sampling strategy	Participants were recruited through convenience sampling from Seoul National University. While no statistical methods were used to determine the sample size beforehand, it is worth noting that the sample size is substantially larger than in previous studies (e.g., Pooresmaeili et al., 2013; Moutsiana et al., 2016; Ho & Schwarzkopf, 2022).
Data collection	Participants were involved in two experiments, an fMRI experiment and a psychophysical experiment. During the fMRI experiment, participants were required to perform a fixation task while their eye movements were being monitored inside the scanner. In the psychophysical experiment, participants performed a circularity discrimination task while their eye movements were being monitored outside the scanner. Only the first author participant was aware of the study hypothesis during data collection. The fMRI and psychophysical experiments were conducted on two separate days.
Timing	The data was collected from February 2016 to June 2016.
Data exclusions	In preparation for analyzing the fMRI data, we had to remove the data from two participants, identified as X010 and X024, due to excessive head movement during the brain imaging process. As a result of this exclusion, we eventually used data from 29 participants for our fMRI data analysis.

Non-participation	For our analysis of psychophysical data, we only included participants who had completed the fMRI experiments. However, we had to exclude three individuals who did not take part in the fMRI experiment due to attrition. Additionally, we excluded data from participants who exhibited significant head motion during brain imaging. With these exclusions, we used data from 27 participants for the psychophysical data analysis.
Randomization	As this study used a within-participant design, we did not need to consider the random assignment of participants into distinct groups. Instead, during a circularity discrimination task, the main task variables (stimulus orientation, aspect ratio of stimulus envelope and stimulus position) were randomized across trials.

Reporting for specific materials, systems and methods

We require information from authors about some types of materials, experimental systems and methods used in many studies. Here, indicate whether each material, system or method listed is relevant to your study. If you are not sure if a list item applies to your research, read the appropriate section before selecting a response.

Materials & experimental systems

n/a	Involved in the study
☒	☐ Antibodies
☒	☐ Eukaryotic cell lines
☒	☐ Palaeontology and archaeology
☒	☐ Animals and other organisms
☒	☐ Clinical data
☒	☐ Dual use research of concern
☒	☐ Plants

Methods

n/a	Involved in the study
☒	☐ ChIP-seq
☒	☐ Flow cytometry
☐	☒ MRI-based neuroimaging

Magnetic resonance imaging

Experimental design

Design type	Retinotopy mapping; Phase-encoded design; Event-related design
Design specifications	uring the functional MRI experiment, nine scans were conducted. Eight of these scans involved traveling waves presented through ring- or wedge-shaped apertures, while the remaining scan involved estimating hemodynamic impulse response (HIRF). Each scan lasted for 216 seconds. The eight traveling-wave scans included two stimulus orientation conditions (radial and tangential) and four traveling-wave aperture conditions (CW, CCW, EXP, and CONT). CW refers to a clockwise rotating wedge, CCW to a counter-clockwise rotating wedge, EXP to an expanding ring, and CONT to a contracting ring. The experiment was conducted in a fixed order, starting with the HIRF scan, followed by CW_c, CW_r, EXP_r, EXP_c, CCW_c, CCW_r, CONT_r, and CONT_c (where "_r" and "_c" indicate the radial and tangential orientation conditions, respectively). Throughout each scan, the stimulus orientation was kept constant to obtain a reliable measure of stimulus orientation-dependent fMRI time-series data.
Behavioral performance measures	To ensure proper fixation during the fMRI experiment, we monitored the observers' performance on a fixation task. They were asked to press a button within 1 second of any reversal in the rotating direction of a pair of small green dots (0.07° in diameter) on a stationary red tangential circle (0.14° in diameter) at the center of the screen. We kept a record of the stimulus history, response (button press) history, and the percentage of correct detections.

Acquisition

Imaging type(s)	functional
Field strength	3T
Sequence & imaging parameters	For anatomical image, a high-resolution T1-w MPRAGE was acquired using a 32-channel head coil (1.9s TR, 2.36 ms TE, 9° flip angle (FA), 1mm isotropic voxel size for 12 observers; 2.4s TR, 3.42 ms TE, 8° FA, 0.8mm isotropic voxel size for 17 observers). For functional image scan, a 20-channel head coil (only the bottom part of a 32-channel head coil) was used to avoid partial blockage of the visual field. GRAPPA, 1.5 s TR, 30 ms TE, 75° FA, 2mm isotropic voxel size, 96 x 80 matrix size, two acceleration factors, and the interleaved slice acquisition order with 62.5 ms inter-slice interval were used. Functional scan follows in-plane T1-w imaging using a 20-channel head coil (MPRAGE, 1mm isotropic voxel, 1.6 s TR, 2.36 ms TE, 9° FA).
Area of acquisition	Whole brain for anatomical image; Early visual areas (the most posterior part at the occipital pole) for functional image

Diffusion MRI ☐ Used ☒ Not used

Preprocessing

Preprocessing software	All functional EPI images were slice-timing corrected and motion-corrected using SPM8 (Friston et al., 1996; Jenkinson et al., 2002). After correction, the functional data from all scans were aligned to the T1-w anatomical image using mrTools analysis package (doi: 10.5281/zenodo.1299483). The cortical surface was constructed using Freesurfer (Dale et al., 1999; Fischl et al., 1999) to create a flattened representation of the occipital lobe. Cortical segmentation of the inner and outer layers of the gray matter was done using Jonas Larsson's SurfRelax. This information was used in mrLoadRet to display a flattened representation of the occipital cortex.
Normalization	Imaging data were not normalized. Each participant's native space was used.
Normalization template	Imaging data were not normalized. Each participant's native space was used.
Noise and artifact removal	N/A
Volume censoring	N/A

Statistical modeling & inference

Model type and settings	Our fMRI time-series analysis relied on a multivariate linear model to estimate the population receptive field (pRF) of the V1, V2, and V3 voxels.
Effect(s) tested	We used a two-tailed paired-sample t-test to examine two things: (1) whether there was a difference in pRF spatial extent between the radial and tangential axes for each of the two stimulus orientation conditions, and (2) whether this difference varied between the two orientation conditions. We measured the degree of anisotropy in pRF spatial extent by calculating the signed difference or divisive contrast between the radial and tangential extents.

Specify type of analysis: ☐ Whole brain ☒ ROI-based ☐ Both

Anatomical location(s)	We manually defined the boundaries between V1, V2, and V3 on the flattened gray matter cortical surface based on the meridian representations by analyzing the temporal phases of fMRI responses to rotating wedge stimuli (Engel et al., 1997).
Statistic type for inference (See Eklund et al. 2016)	N/A
Correction	N/A

Models & analysis

n/a	Involved in the study
☒	☐ Functional and/or effective connectivity
☒	☐ Graph analysis
☐	☒ Multivariate modeling or predictive analysis

Multivariate modeling and predictive analysis	We used a multivariate linear model to estimate the population receptive field(pRF) of the V1, V2, and V3 voxels. Specifically, we fitted a 2D Gaussian model to the fMRI time series for the purpose of determining the center position and spatial extent parameters of the pRF.
